# Editorial: Advances in Equine Dental and Sinonasal Disorder Research

**DOI:** 10.3389/fvets.2022.852087

**Published:** 2022-03-07

**Authors:** Padraic M. Dixon, Richard J. M. Reardon, Lieven Vlaminck

**Affiliations:** ^1^Independent Researcher, Edinburgh, United Kingdom; ^2^The Royal (Dick) School of Veterinary Studies and The Roslin Institute, The University of Edinburgh, Edinburgh, United Kingdom; ^3^Department of Large Animal Surgery, Anaesthesia and Orthopaedics, Faculty of Veterinary Medicine, Ghent University, Merelbeke, Belgium

**Keywords:** equine sinonasal disorders, equine sinusitis, equine sinus osteitis, equine sinonasal imaging, equine dentistry, equine dental fractures

This collection in the veterinary dentistry and oromaxillofacial surgery speciality section of Frontiers in Veterinary Science presents 10 peer-reviewed research articles that investigate many important topics in equine dentistry and sinonasal disorders.

Three of the papers used computed tomography (CT) to investigate equine sino-nasal disorders that are often caused by dental disease.

A study by Dixon, Puidupin et al. showed that horses with sinus disease have significant inflammation of the bones adjacent to the paranasal sinuses. This sinusitis-related osteitis can explain the presence of epiphora, soft tissue facial swellings and the increased maxillary bone radionucleotide uptake in sinusitis cases. Osteitis and distortion of the maxillary septal bulla or infraorbital canal can also explain the difficulty in fenestrating the maxillary septal bulla in some horses with sinus disease.

A study of 300 horses with sinus disease that all underwent CT examinations, included 100 consecutive referred cases from three different equine referral centers (Dixon, Barnett, et al.). This study identified dental sinusitis and chronic primary sinusitis as the main types of sinus disease. The rostral maxillary sinus (94.7% involvement) and the ventral conchal sinus (87% involvement) were the most commonly affected compartments. Notably, this study also showed involvement of the nasal conchal bullae in 56% of horses with sinus disease.

Sinonasal drainage obstruction of their normal mucus secretions can occur in miniature horse breeds, possibly due to developmental distortion of their sino-nasal bony structures (Vlaminck et al.). This study by Vlaminck et al. described the diagnosis of 7 such cases, including by CT, in young (mean age 2.2 years) American miniature horses and miniature Shetland ponies. Their successful treatment was by fenestration of the dorsal conchal sinus into the nasal cavity *via* nasofrontal osteotomies followed by temporary catheterisation of the sino-nasal fistula to maintain patency.

A further CT study by Liuti et al. examined the angulation and age-related dental drift of the rostral (second premolar tooth; Triadan 06) and caudal (third molar tooth; Triadan 11) mandibular and maxillary equine cheek teeth in horses of different ages. The study showed much higher angulation in the mandibular vs. the maxillary teeth, and in the third molar teeth (Triadan 11 s) vs. the second premolar teeth (Triadan 06s). The angulation of most teeth did not decrease with age contrary to expectations. The caudal (distal) angulation of the second premolar teeth (Triadan 06) clinical crowns might indicate that distal drift occurs in these teeth, but in fact all teeth drifted mesially with age. Further understanding of age-related dental drift and dental angulation may help elucidate the causes and treatment of the very common and painful disorder of equine cheek teeth diastema.

A retrospective study by Gergeleit and Bienert-Zeit found clinically significant post-extraction complications following 6.6% (*n* = 20) of 302 mandibular cheek tooth extractions. Horses developing complications were younger than those not developing complications and the Triadan 07s and 09s were the most commonly affected teeth. Alveolar sequestration was the most prevalent complication, occurring in 18/20 horses, with complete alveolar sequestration occurring in some cases. Post-extraction mandibular fistula formation occurred in 5/20 cases and mandibular abscessation in 4/20 cases. All cases were successfully treated, including by sequestrectomy and wound debridement with some cases taking up to 5 months to resolve.

There has been debate on the presence and frequency of communications between the discotemporal joint (DTJ) and the discomandibular joint (DMJ) in normal equine temporomandibular joints (TMJ). A study by Pimentel and Carmalt examined for the presence of communications between these two compartments in 20 normal horses. No physiological communication between the two compartments was identified. However, 2/40 joints had an acquired communication between the DTJ and DMJ. This finding is clinically relevant when considering the use of local anesthesia or medication in these compartments.

Abnormalities of equine maxillary cheek teeth infundibula are common, mainly varying degrees of developmental cemental hypoplasia with later acquired caries that may predispose to fracture or apical infection of these teeth. This article by Pearce and Brooks is the first peer-reviewed study on the long-term clinical and oral endoscopic finding in horses undergoing infundibular caries treatment. The described infundibular restoration technique was found to be safe and most restorations lasted for many years (including for over 10 years in some). Only 1.3% of treated teeth later developed fractures or apical infection thus supporting the value of this technique.

Three studies examined non-traumatic equine cheek teeth fractures: fissure fractures (*n* = 2) and gross fractures (*n* = 1).

An *ex vivo* study by Pollaris et al. assessed whether the presence of fissure fractures or other anatomical features influenced the development of gross fractures when mechanical pressure was applied to the occlusal surface of these teeth. Resistance to fracture was found to vary between different sites on the occlusal surface of healthy equine cheek teeth, and the presence of fissures further decreased fracture resistance.

A 3-year longitudinal study by Pollaris et al. of 36 horses with cheek teeth fissure fractures assessed for long-term changes in these teeth, including determining if any of these fissure fractures later converted to gross fractures. The study found that over time, the fissure fractures could remain unchanged, disappear, become longer, shorter, change in configuration or in color. Gross fractures (all partial crown fractures, with none resulting in direct communication with the pulp) were found to be more likely when: fissure fractures were present, in mandibular (rather than maxillary) teeth and on the lingual (rather than the buccal) side of the tooth.

A study of 300 horses with 486 non-traumatic gross cheek teeth fractures by Dixon, Kennedy et al. showed the maxillary teeth to be most commonly affected, including 1st and 2nd pulp horn (“slab”) fractures and caries-related infundibular fractures. Mandibular cheek teeth 1st and 2nd pulp fractures were the most common mandibular cheek teeth fractures. Almost half of affected horses had no clinical signs, the others mainly having signs caused by apical infection or oral pain. The stable remnants of fractured teeth were not extracted in 60% of cases.

It is hoped that this collection of studies will encourage further basic and clinical equine dental and sinonasal research. The increasing number of standing CT systems will allow further studies of these anatomically complex areas with multiple overlapping structures, that until recently have had restricted two-dimensional imaging by radiography. The increasing use of quality oral endoscopes will allow further and more detailed oral examinations such as the recognition cheek teeth fissure fractures and allow further studies on the progression or regression of such lesions, as found in one of the above studies. Finally, the increasing numbers of American and European Board Specialists in equine dentistry will hopefully increase the pool of researchers in this field.

## Author Contributions

This editorial was written by PD and edited by RR and LV. All authors contributed to the article and approved the submitted version.

## Conflict of Interest

The authors declare that the research was conducted in the absence of any commercial or financial relationships that could be construed as a potential conflict of interest.

## Publisher's Note

All claims expressed in this article are solely those of the authors and do not necessarily represent those of their affiliated organizations, or those of the publisher, the editors and the reviewers. Any product that may be evaluated in this article, or claim that may be made by its manufacturer, is not guaranteed or endorsed by the publisher.

